# 

**Published:** 1880-10

**Authors:** 


### CONTENTS:

ART. I.—The Importance of Direct Sunlight in the Operating Room.
By J. N Farrar, M D., D. D. S.............241
ART. II.—General Care of the Mouth for Patients Under Twenty Years of Age.
By Dr. L. C. Taylor............259
ART. III.—Bromide of Ethyl.............266
ART. IV.—Articulating Artificial Teeth. By W. E. Driscoll. D. D. S.............269
ART. V.—Diseases of Wild Animals. By Prof. Jean Vilian.............272
ART. VI.—Rapid Breathing as a Pain Obtunder.............275
ART. VII.—The Position of the Dentist.............277

### EDITORIAL, ETC.

The Forty-first Annual Course of Instruction in the Baltimore College of Dental Surgery.............279

### BIBLIOGRAPHICAL.

Nasal Catarrh. By Prof. Martin Coomes.............281
Common Mind Troubles and the Secret of a Clear Head.
By J. M. Granville, M. D.............281
Report of Health Officers of District of Columbia.............282
Rowell's American Newspaper Directory.............282
The Skin in Health and Disease. By L. Duncan Bulkley, M. D.............282
School and Industrial Hygiene. By D. F. Lincoln. M. D.............283

### MONTHLY SUMMARY.

Watching the Pulse During the Administration of Chloroform.............283
Use of Chloroform in Diseases of the Heart.............285
Tragedy in a Dental Office.............285
Apparent Death from Asphyxia.............286
A New Anæsthetic.............287
Swallowing False Teeth.............287
Classic Dentistry.............288
Is Salycilic Acid Injurious to the Teeth.............288
The Temperature of the Breath.............288

